# Metacarpal Fractures, Management Techniques, and Outcomes in Our Center

**DOI:** 10.7759/cureus.17828

**Published:** 2021-09-08

**Authors:** Alina Fatima, OWais Ahmed, Mehtab Ahmed, Mirza Shehab A Beg, Arooba Batool, Muhammad Muneebullah Siddiqui

**Affiliations:** 1 Plastic and Reconstructive Surgery, Liaquat National Hospital and Medical Collage, Karachi, PAK; 2 Plastic and Reconstructive Surgery, Liaquat National Hospital and Medical College, Karachi, PAK; 3 Community Health Sciences, Agha Khan University Hospital, Karachi, PAK

**Keywords:** metacarpal fracture, fracture fixation, k-wire, grip strength, screw, plates, range of movement

## Abstract

Introduction/background

Metacarpal fractures comprise approximately 35.5% of cases in daily emergencies, mostly due to road traffic accidents (RTA), fall, and assault. The classification is based on the site and pattern of fracture. High-level evidence is lacking for the management of metacarpal fractures. The primary goals of treatment are to achieve acceptable alignment, stable reduction, strong bony union, and unrestricted motion. It can be managed by non-operative methods like close reduction and splintage. Operative management will be required if there is shortening, rotation, and angulation in different planes including close reduction and fixation with percutaneous intramedullary pining/k-wires and open reduction and fixation with screws, plates (compression/locking), and external fixators. This study was done to compare the efficacy of k-wire, screws, and plates in the management of metacarpal fractures and their outcomes based on their union, postoperative pain, range of movement, and grip strength in a tertiary care center, i.e., Liaquat National Hospital and Medical College.

Methods

It was a retrospective study conducted at the Department of Plastics and Reconstruction Surgery, of a tertiary care hospital. A total of 113 patients who were operated upon for metacarpal fracture were included in the study (open/close) without soft tissue loss or tendon injury, were divided into three groups according to the technique of fracture fixation, i.e., group 1 (k-wire), group 2 (screw), and group 3 (plates). The data like post-operative pain (visual analog scale, VAS) and radiological evidence of union were extracted from the registry. All the patients were called for follow-up in the outpatient department. Out of 113, 97 patients showed up for follow-up and were examined by a hand surgeon, and range of movement (goniometer) and grip strength (sphygmomanometer method) were assessed.

Results

A total of 97 patients were included in the study (male 66%, female 34%). Group 1 (K-wire) includes n = 61 (62.9%), group 2 (screw) n = 15 (15.5%), and group 3 (plate) n = 21 (21.6%). The mean follow-up time was 12 + 2 weeks after the surgery for post-operative pain and radiological evidence of union while 24 + 6 months for a range of movement and grip strength. Less post-operative pain was noted in group 1 patients while no significant difference was noted in the evidence of radiological union in all groups. Range of movement was better in group 1 patients (89.74 + 0.750) than in group 2 (80 + 0.37°) or group 3 (80.2 + 0.62°). The grip strength (compared to the normal contralateral hand) was normal in the majority of the patients in group 1, i.e., 94% while it was 80% in group 2 and 82% in group 3.

Conclusion

The significance of these reported findings suggests that open reduction and internal fixation with screw or plate might be a less preferable surgical technique in comparison to k-wire fixation in the treatment of a metacarpal fracture.

## Introduction

Metacarpal fractures comprise approximately 35.5% of cases in daily emergencies. Causes may be due to road traffic accidents (RTA), fall, and assault. If left untreated, it may result in structural deformity and may result in stiffness if it is over-treated [1-3]. It includes around 18-44% of total hand fractures [4], and the fifth metacarpal is the most commonly fractured among all [5].

The metacarpal fractures can be classified based on the site and pattern of fracture. As per the anatomic site, it could be a metacarpal head, neck, shaft, or base fracture [1]. The description of the pattern of fracture is the same as of other long bones that may be open or close, intra-articular or extra-articular, and could be oblique, spiral, transverse, or comminuted [6].

The metacarpal head and base are primarily cancellous bones, and the metacarpal shaft is primarily cortical bone [6]. The blood supply of the metacarpals is rich and in general, enables the metacarpal to heal well after a fracture. The rate of healing is more efficient in the more cancellous bone of the metacarpal head and base compared with a shaft fracture, which involves the cortical bone [6]. The metacarpal is attached to carpal bones at their bases forming the carpometacarpal joint (CMCJ). The index and middle CMCJ are relatively stiff and immobile as compared to the ring and little CMCJ having 15° of flexion and 25° of extension [7]. The metacarpals are stabilized by numerous tendinous and ligamentous structures, that must be kept in mind when performing fixation of metacarpal fractures, that is deep transverse metacarpal ligament holds the metacarpal heads, dorsal and palmar interossei could be responsible for dorsal angulation of the fractured metacarpal as they arise from its shaft.

High-level evidence is lacking for the management of metacarpal fractures. The majority of studies suggest that the fractures that are more ulnar and or more distal with minimal to no soft tissue involvement can be best managed by non-operative methods, i.e., the close reduction and splintage [1,6]. Metacarpal neck fractures are the most common, closed reduction can be accomplished, for fracture of the neck of the fifth metacarpal; the close reduction can be achieved with the Jahss maneuver [8]. For metacarpal shaft fractures, simple closed reduction and immobilization may be used to treat stable fractures. Metacarpal head fractures can also be managed non-operatively if the joint involvement is <20%. Patients are immobilized three to four weeks in an ulnar or radial gutter splint maintaining metacarpophalangeal joint (MCPJ) flexion. If the reduced fracture is unstable, percutaneous pinning can be done [9].

Operative management will be required if there is shortening, rotation, and angulation in different planes [1]. Other indications for operative intervention includes unstable fracture, irreducible, open fractures, fractures with segmental bony loss, multiple fractures, fractures associated with significant soft-tissue injury, more than 25% involvement of the articular surface of the metacarpal head, displaced fractures of the metacarpal base with dislocation or subluxation of the CMCJ [10].

The primary goals of treatment are to achieve acceptable alignment, stable reduction, strong bony union, and unrestricted motion. To address the metacarpal fracture, multiple techniques are described in the literature including close reduction and fixation with percutaneous intramedullary pining/k-wires and open reduction and fixation with screws, plates (compression/ locking), and external fixators [11]. Each technique has its pros and cons related to the type and site of fracture and involvement of soft tissue.

The complications of metacarpal fixation are mainly related to the type of injury and surgical technique opted for fixation. General complications include malunion, nonunion, problems with soft tissue healing leading to joint pathology (contractures or hyperextension deformity), tendon tethering, and intrinsic shortening [3]. The MCPJ is at the greatest risk for altered mobility after head and neck fractures. All these complications ultimately lead to stiffness.

In a study, Kelsch and Ulrich concluded that stabilization of metacarpal with closed reduction and fixation with intramedullary k-wire produces good long-term functional results [12]. However, a high level of evidence is not available in the literature suggesting one technique over another. The reported complications in terms of post-operative stiffness and restricted hand movements are related to open reduction and internal fixation while close reduction and fixation with k-wire results in superficial infections managed conservatively [13].

The purpose of this retrospective study was to compare the efficacy of k-wire, screws, and plates in the management of metacarpal fractures and their outcomes based on their union, postoperative pain, range of movement, and grip strength in a tertiary care hospital.

## Materials and methods

It was a retrospective study conducted at the Department of Plastics and Reconstruction Surgery, of a tertiary care center, i.e., Liaquat National Hospital and Medical College, Karachi, Pakistan. A search of patient records from the hospital information management system (HIMS) and outpatient registry was done and extracted data of 180 patients were operated upon for metacarpal fractures from July 2016 to June 2019. For homogeneity of the study, patients with closed and open fracture (without soft tissue loss or concomitant tendon injury) of metacarpal treated with k-wires, screws, and plates were included. The reason for choosing a specific treatment was surgeon selected and was not mentioned in the records. All the patients were sent to a qualified hand physiotherapist after six weeks of surgery.

The patients under 18 years of age were excluded as they have developing bones with different remodeling capacities and older than 55 years of age due to their decreased functional demands. Patients with previous injuries of the ipsilateral hand or wrist, concomitant injuries of the same limb, and who were lost to follow-up within 12 weeks of the surgery were also excluded from the study. From a total of 180, 113 patients fulfilled both the inclusion and exclusion criteria. The majority of them were operated on by closed reduction and fixation with percutaneous k-wires.

Ethical review was taken from the institutional ethical review board. All patients were contacted via phone and invited to participate in the study and to come for the follow-up visit in our outpatient department. Ninety-seven patients were presented in the outpatient department for follow-up and were examined by a hand surgeon after informed consent. The patients were divided into three groups according to the type of surgical technique acquired to fix the metacarpal fracture, group 1 (k-wire), group 2 (screw), and group 3 (plate). The range of movement at the MCPJ and grip strength were measured at the time of follow-up. The range of movement at the MCPJ was measured with a goniometer [14]. A goniometer was placed at the MCPJ and the angle was noted on complete flexion of the joint (measured in degrees). The average angle for a normal hand, when measured with a goniometer, should be 90° on full flexion [14]. The grip strength was assessed using a modified sphygmomanometer [15].

For measuring the grip strength position of the participant’s arm was kept in the position that is he/she remain seated on a chair with supported trunk and feet, shoulder adducted, elbow flexed at 90°, forearm in the neutral position, and wrist with 0 to 30° extension [16]. The sphygmomanometer was calibrated and pre-inflated at 20 mmHg and the patient was asked to press the cuff to his/her maximum grip strength and pressure gauge reading was noted, considering increments on 2 mmHg [17,18]. The reading was compared with the other hand and checked whether it is normal (difference <15 mmHg) or decreased (difference >15 mmHg).

The data regarding pain at rest and at the activity (assessed by using the visual analog scale (VAS)) and fracture union (assessed with the help of an X-ray by observing the loss of fracture lines and cortical continuity) were extracted from the registry. These data were being documented in the registry at 12 to 14 weeks after the surgery. These data were extracted from the registry regarding basic demographics including age, occupation, hand dominance, fracture type (open/close), fracture site on metacarpal (head/neck/shaft/base), fracture pattern (simple/comminuted), fixation techniques (k-wire/screw/plate), and complications related to the procedure and were documented on a proforma.

Data analysis

Data were entered and analyzed through a statistical package for social sciences (SPSS) version 25 (IBM Corp., Armonk, NY). The mean + SD were calculated for continuous variables, i.e., age, pain on VAS, angle depicting the range of movement, and grip strength. Frequency/percentage were assessed for qualitative variables, i.e., gender, occupation, hand dominance, mechanism of injury, type of fracture, site, pattern, type of fixation technique, and radiological evidence of fracture union. Pie and bar charts were utilized for the graphical display of results. Mean difference of pain, angle depicting range of movement, and grip strength in all the techniques were compared using ANOVA (p-value <0.05).

## Results

Demographics

**Figure 1:**
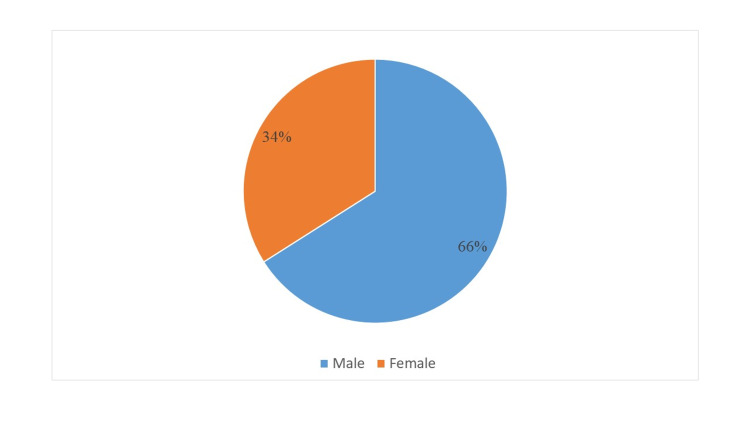
Gender (n=97)

**Figure 2:**
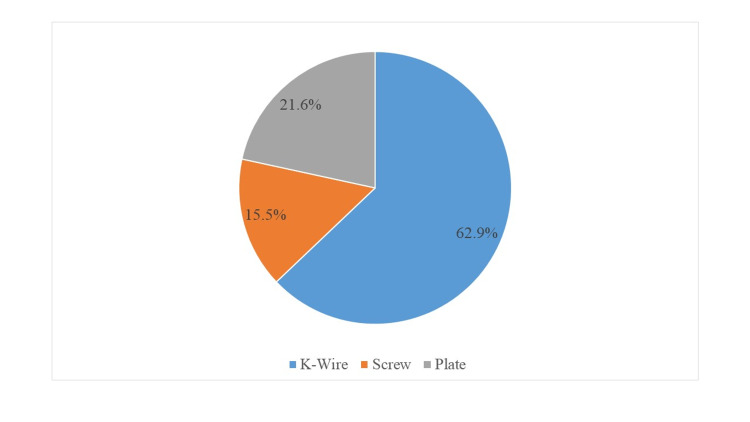
Fracture fixation techniques (n = 97)

A total of 97 patients were included in the study (male 66%, female 34%; Figure 1) and were divided into three groups on the basis of type of surgical technique used for metacarpal fractures fixation group 1 (k-wire) n = 61 (62.9%), group 2 (screw) n = 15 (15.5%), and group 3 (plate) n = 21 (21.6%; Figure 2).

The mean age of patients of group 1 was 32.6 + 8.2 years [male 41 (67.2%), female 20 (32.8%)], group 2 was 32.7 + 6.98 years [male 9 (60%), female 6 (40%)], and group 3 was 32.5 + 8.14 years [male 14 (66.7%), female 7 (33.3%)] (Table 1). Majority of the patients were office workers in group 1 (49%), while majority were manual labours in group 2 (40%) and in group 3 (52%). Groupwise distribution of patients occupation, hand dominance (right/left), fracture type (open/close), and fracture pattern (simple/ comminuted) is presented in Table 1.

**Table 1:** Demographics

		Group 1 K (n = 61)	Group 2	Group 3
Age	Years (SD)	32.61 (8.2)	32.7 (6.9)	32.5 (8.14)
Gender	Male	41 (67.2%)	9 (60%)	14 (66.7%)
	Female	20 (32.8%)	6 (40%)	7 (33.3%)
Occupation	Office worker	30 (49.2%)	5 (33.3%)	9 (42.9%)
	Manual worker	24 (39.3%)	6 (40%)	11 (52.4%)
	Student	6 (9.8%)	4 (26.6%)	1 (4.8%)
	Other	1 (1.6%)	0 (0.0%)	0 (0.0%)
Hand dominance	Right	42 (68.9%)	11 (73.3%)	18 (85.7%)
	Left	19 (31.1%)	4 (26.7%)	3 (14.3%)
Fracture type	Open	18 (29.5%)	6 (40%)	7 (33.3%)
	Close	43 (70.5%)	9 (60%)	14 (66.7%)
Fracture pattern	Simple	25 (41%)	10 (66.3%)	14 (66.6%)
	Comminuted	36 (59%)	5 (33.3%)	7 (33.3%)

**Figure 3:**
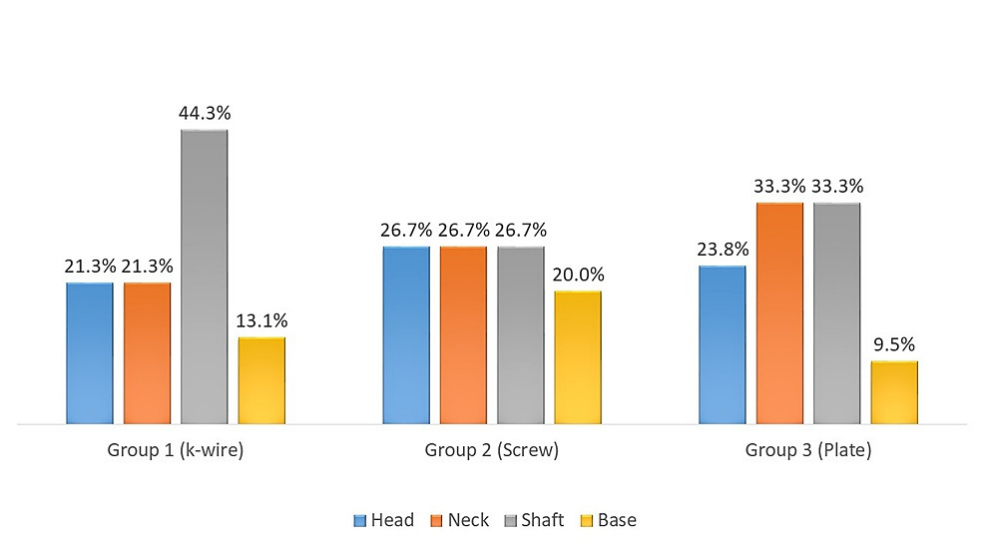
Fracture site at the metacarpal

The site of fracture in majority of the patients in group 1 was metacarpal shaft, i.e., n = 27 (44.3%), head 13 (21.3%), neck 13 (21.3%), and 8 (13.1%) at the base; in group 2, it was shaft in 4 (26.7%), head 4 (26.7%), neck 4 (26.7%), and base 3 (20%), while in group 3, was shaft in 7 (33.3%), neck 7 (33.3%), head 5 (23.8%), and base in 2 (9.5%; Figure 3).

Clinical outcomes

The mean follow-up time was 12 + 2 weeks after the surgery for all the patients to assess pain (VAS) and radiological evidence of fracture union, while it was 24 + 6 months for grip strength and angle of MCPJ compared with normal hand.

**Figure 4:**
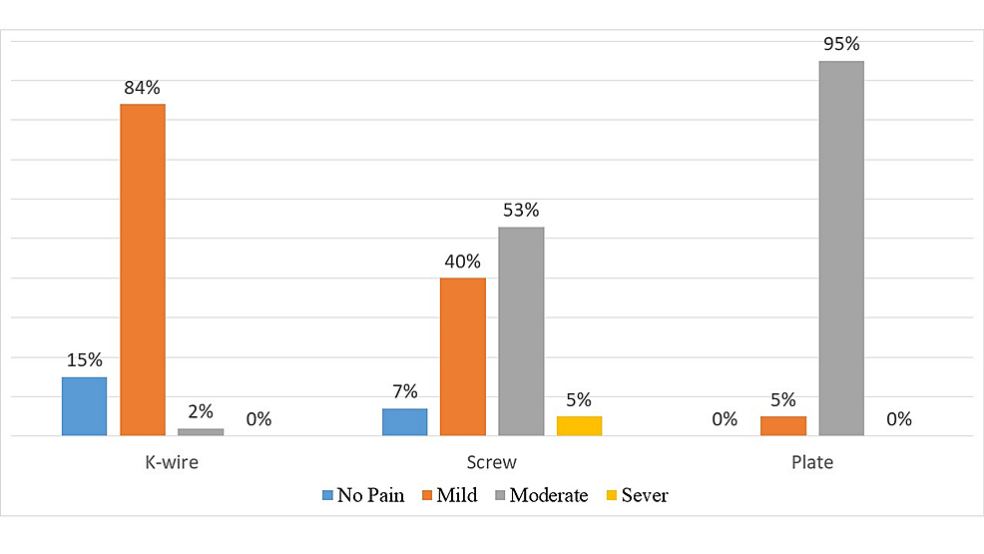
Mean pain score on the activity as per VAS (12 - 14 weeks post-op)

At 12 + 2 weeks, there was no pain at rest in patients of all the groups but on the activity, the mean pain score in patients of group 1 (1.23 + 0.76) was lower than that of group 2 (2.4 + 1.2) and group 3 (3.33 + 0.57) patients as per VAS. It is categorized as no pain, mild, moderate, and severe on VAS. A majority of patients in group 1 experienced mild pain (84%), in group 2, it was mild in 40% and moderate in 53% while the majority of patients in group 3 experienced moderate pain on activity (95%) (Figure 4).

**Figure 5:**
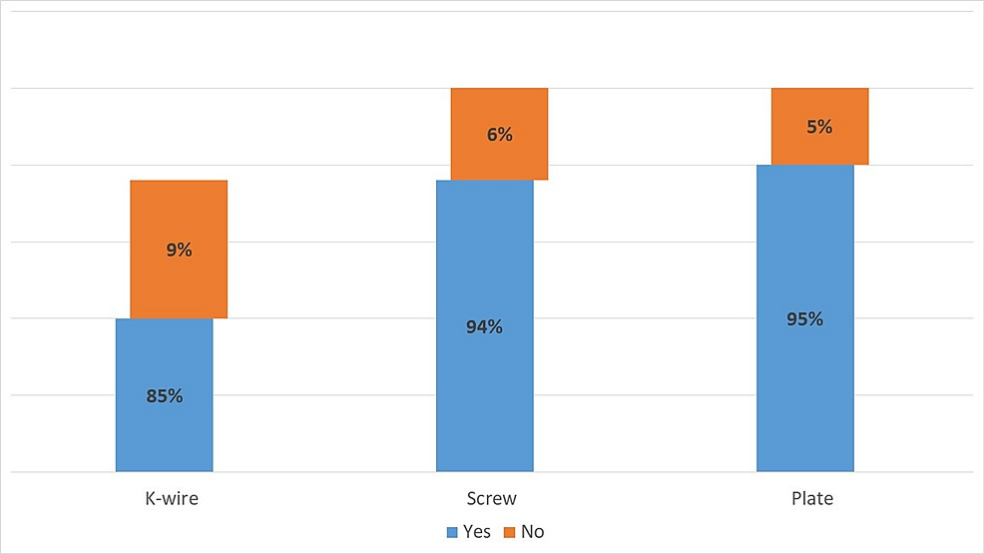
Evidence of radiological union (12 to 14 post-op weeks)

The evidence of radiological union was noted in most of the patients in all groups slightly better in groups 2 and 3 patients as compared to group 1 patients, that is 85% in group 1, 94% in group 2, and 95% in group 3 patients (Figure 5).

The patients were examined at 24 + 6 months post-operatively for the mean angle of MCPJ; it was 89.74 + 0.75 0 in group 1 patients while 80 + 0.370 in group 2 patients, and 80.2 + 0.620 in group 3 patient, that is better in group 1 patients as compared to group 2 and 3 patients. The mean angle of MCPJ was nearly normal in patients in all three groups when compared to the contralateral normal hand. Statistical test ANOVA was applied to compare the means of the angle of MCPJ and it was found significant (p-value <0.05). The grip strength was measured by using a modified sphygmomanometer and compared to the contralateral normal hand and it was found that it was normal in 94% of patients of group 1 (mean 227 + 25.7 mm of Hg), 80% in group 2 (mean 208 + 26.6 mm of Hg), and 82% of patient of group 3 (mean 216 + 28.3 mm of Hg), which was less than that of group 1 patients which were statistically significant (p-value 0.021) when means were compared using statistical test ANOVA.

None of the patients in each group suffered any serious complications except five patients in group 1, who developed pin tract infections that were managed conservatively with dressings and oral antibiotics.

## Discussion

Metacarpal fractures comprise approximately 35.5% of cases in daily emergencies. Common causes of these fractures include RTA, fall, and assault. If left untreated, it may result in a deformity that can cause severe disability and affect daily routine activities and may result in stiffness if over-treated [1-3]. It includes around 18-44% of total hand fractures [4]. The fifth metacarpal is the most commonly fractured among all the metacarpals [5]. The metacarpal is just like the long bone of the upper and lower limb, starting proximally from the CMCJ ending to the metacarpophalangeal joint (MCPJ). The index finger metacarpal is the longest and the most stable. The index and long fingers have 0° motion at their CMC joint [6]. The ring and small finger metacarpals are of decreasing length and increasing CMC joint mobility that is 15° and 25° [1,2].

The mobility of the ring and small metacarpals depends on their articulation with the modified saddle joint of the hamate. In contrast, the stability of the bases of the index and long metacarpals is due to the taut ligaments and bony contact with the carpals. The thumb is the shortest and the most mobile metacarpal. The thumb CMC is a biconcave saddle joint. Articular congruity and strong capsular ligaments provide stability for the highly mobile thumb to oppose the other digits and to make better contact with objects during grasp and pinch. Distally, the deep transverse metacarpal ligament and the volar plates link the metacarpal heads to enhance the strength of these skeletal arches. The intrinsic and extrinsic muscles provide added support to the skeletal arch systems [6].

The metacarpal fractures can be classified based on the site and pattern of fracture. As per the anatomic site, it could be a metacarpal head, neck, shaft, or base fracture [1]. Simple closed reduction and immobilization may be used to treat stable fractures. Percutaneous pinning may be added to stabilize the reduced metacarpal fragments to the adjacent metacarpal axially or obliquely to establish fixation. Open fixation is indicated in the event of multiple metacarpal fractures, inadequate reductions, and fractures that remain unstable despite pinning. In addition to Kirschner wires, plate fixation, wires, and lag screws are excellent techniques for internal fixation [1].

Metacarpal head fractures can be treated non-operatively or with closed reduction and internal fixation if the joint involvement is <20%. In the cases in which the joint articular surface has >20% involvement or displaced ligament avulsion fractures are present, open reduction is indicated [2]. Metacarpal neck fractures, the most common of all metacarpal fractures, usually involve the small and ring fingers and commonly are referred to as Boxer’s fractures. For open wounds with signs of infection, treatment should be delayed until control of the infection. Percutaneous pinning is employed if the reduced fracture is unstable. Open reduction is reserved for fractures that cannot be reduced manually [1].

Multiple management techniques have been described to provide optimal treatment of unstable metacarpal fractures including splintage, initial immobilization in the intrinsic plus position, internal fixation with k-wires, screws, plates, and external fixation. The goal of treatment is to correct rotational alignment, form a strong bony union, achieve unrestricted motion prevent stiffness, and maximize function. K-wires have the lowest bending strength and are best for fracture fixation where plates or screws may be difficult to place and to maintain reduction of dislocated metacarpals. However, complications vary according to the type of fixation and there is an increased association of pin tract infection and problems due to protruding ends of k-wire [19].

Many studies are showing the satisfactory result of metacarpal fracture managed by percutaneous k-wiring for fracture fixation. A study reported good functional outcomes for percutaneous pinning with no functional impairment in k-wire treated patients and our study showed the same results [13]. Another study by Lee et al. concluded that k-wires facilitate early hand mobilization, correct the deformity, and provide good clinical and radiographic outcomes [19]. As in our study, the functional outcome in terms of postoperative pain and grip strength is much better than that of open methods including screws and plates. Another study by Kelsch and Ulrich [12], showed that intramedullary k-wire for fixation is generally believed to be the least invasive technique with maximum long-term function. The remarkable long-term clinical results are because the gliding tissue around the fracture will not be affected at all by the surgical procedure [13]. There were a few limitations of our study including the difference in the number of patients in each group, limited sample size, and there were no specific criteria for selecting the type of surgical technique as it was a retrospective study.

In our study, we have found that k-wires as compared to screws and plates are versatile, easily available, cheap, surgeon friendly. They provide an adequate range of movement, grip strength, and overall good functional outcome when assessed at 12-14 weeks post-operation. We suggest further prospective clinical trials to establish the proper guideline for selecting an appropriate surgical technique to adopt for the fixation of metacarpal fracture and to see the functional outcomes.

## Conclusions

The screw and plate fixation is done by using an open approach with extensive dissection which increases the morbidity in terms of post-operative pain, stiffness, and scar formation that is not the case with k-wire fixation as the procedure is done via percutaneous approach. The screws and plates appear bulky and are felt subcutaneously that can have poor patient compliance and often needs removal in the operation theater further adding the cost. On the other hand, the k-wires are removed once the healing is achieved; the procedure is usually performed in a clinic setting and does not need to go to the operation room.

The significance of our reported findings suggests that open reduction and internal fixation with screw or plate might be a less preferable surgical technique in comparison to k-wire fixation (percutaneous pinning) in the treatment of a metacarpal fracture as it is readily available, cost-effective, requires less operative time, and is relatively easy to perform. It also has better postoperative functional outcomes in terms of postoperative pain, grip strength, and range of movement at the MCPJ.
